# Surface Quality Assessment after Milling AZ91D Magnesium Alloy Using PCD Tool

**DOI:** 10.3390/ma13030617

**Published:** 2020-01-30

**Authors:** Ireneusz Zagórski, Jarosław Korpysa

**Affiliations:** Department of Production Engineering, Mechanical Engineering Faculty, Lublin University of Technology, 20-618 Lublin, Poland; j.korpysa@pollub.pl

**Keywords:** magnesium alloy, PCD tool, high-speed dry milling, surface roughness, surface topography

## Abstract

Surface roughness is among the key indicators describing the quality of machined surfaces. Although it is an aggregate of several factors, the condition of the surface is largely determined by the type of tool and the operational parameters of machining. This study sought to examine the effect that particular machining parameters have on the quality of the surface. The investigated operation was the high-speed dry milling of a magnesium alloy with a polycrystalline diamond (PCD) cutting tool dedicated for light metal applications. Magnesium alloys have low density, and thus are commonly used in the aerospace or automotive industries. The state of the Mg surfaces was assessed using the 2D surface roughness parameters, measured on the lateral and the end face of the specimens, and the end-face 3D area roughness parameters. The description of the surfaces was complemented with the surface topography maps and the Abbott–Firestone curves of the specimens. Most 2D roughness parameters were to a limited extent affected by the changes in the cutting speed and the axial depth of cut, therefore, the results from the measurements were subjected to statistical analysis. From the data comparison, it emerged that PCD-tipped tools are resilient to changes in the cutting parameters and produce a high-quality surface finish.

## 1. Introduction—State of the Art

Magnesium alloys are the lightest metals used for construction purposes. Due to their low specific weight and high strength, their range of industrial applications is constantly increasing. The favorable properties also include recyclability, damping capacity, and good casting properties [1,2]. These qualities are particularly desirable in aerospace and automotive industries, which are constantly striving for ever lighter structures. Magnesium alloys, which belong to the group of light metals, are therefore suitable for whenever weight reduction is crucial. The increasing share of this group of materials in various industries increases the need for continuous development and improvement of machining processes. The requirements for manufactured elements are constantly increasing, so it is reasonable to strive to improve the quality of manufactured elements [3,4,5].

The roughness of the surface is the most commonly employed indicator of surface quality. However, to date, the scientific investigations have limited the evaluation of the post-machining condition of the surface to the analysis of its 2D surface roughness parameters. In addition, of the roughness parameters the most widely considered are Ra (an arithmetic mean profile deviation) and Rz (a cusp height of the profile). For accuracy, a wider range of 2D surface and 3D area roughness parameters should be included in the description. In machine construction, surfaces are expected to perform predictably, i.e., to exhibit a certain set of features or utilitarian properties (fatigue strength, tribological, or adhesive properties). Their prediction is important in the context of sliding friction phenomena, and thus of abrasive wear. Currently, the geometrical state of the surface is regarded as equally important as the dimensional and shape accuracy. However, due to certain problems with providing a clear definition of the relationships between the technological and utilitarian qualities of surfaces, it is difficult to directly infer on the operational and functional properties of surfaces from the 2D surface and 3D area roughness parameters of machined surfaces [6].

The benefits of machining with PCD-tipped tools have been confirmed, e.g., by Kuczmaszewski et al. [7]. In the study, three types of tools were used on AZ31 and AZ91HP magnesium alloy substrates: a TiAlN-coated carbide milling cutter, a PCD-tipped milling cutter, and a Kordell milling cutter. It was found that the surface roughness parameters did not show a marked drop after machining at increased cutting speeds (at v_c_ = 400–1200 m/min) and the best performing tool was the PCD-tipped tool–Ra = 0.2–0.4 μm and Rz = 1.5–2.8 μm; subsequently, for the Kordell carbide tool the values were Ra = 1.2–2.8 μm and Rz = 6.0–12.0 μm, and for the TiAlN-coated tool–Ra = 3.0–5.4 μm and Rz = 12.0–26.0 μm. Similarly, when the feed per tooth was adjusted (f_z_ = 0.05–0.30 mm/tooth), the lowest roughness, Ra = 0.2–0.5 μm and Rz = 1.5–3.0 μm, was observed in the case of the PCD tool, next the Kordell tool, Ra = 0.5–3.6 μm and Rz = 2.0–17.0 μm, and the TiAlN-coated cutter: Ra = 1.0–7.6 μm and Rz = 5.0–36.0 μm.

Guo and Salahshoor [8,9] analyzed the machinability of Mg-Ca biodegradable magnesium alloys during dry milling with a PCD-tipped face milling head carried out in the v_c_ range up to 2800 m/min. By modifying the cutting speed, the best surface roughness (Ra) obtained amounted to approximately 0.4 μm. A similar relationship was observed in the case of the depth of cut (a_p_) adjustment. However, it was the feed per tooth that was shown to most affect the surface quality: the Ra parameter changed from 0.2 μm at feed f_z_ = 0.05 mm/tooth to 0.78 μm at feed f_z_ = 0.4 mm/tooth. For comparison, when applying finish burnishing at F = 400–800 N, the value of Ra was in the region of 0.6 μm.

Analogous relationships were discovered by Desai et al., who described the machining of the Mg-Ca1.0 alloy with a multi-edge milling cutter with the diamond-like carbon coating (DLC) [10]. The roughness of the surface was assessed with the Ra parameter, which was shown to decrease to the range of 0.079–0.159 μm, depending on the specific cutting data: the cutting speed range v_c_ = 300–600 m/min, the feed per tooth f_z_ = 0.0125–0.125 mm/tooth and the axial depth of cut a_p_ = 0.05–0.50 mm. The surface roughness was observed to decline at higher cutting speeds and increase at higher feeds; nevertheless, the average roughness remained very low.

Biodegradable Mg alloys have also been subjected to testing by Qiao et al. [11]. The MgCa0.8 alloy surfaces were machined with a milling cutter head with uncoated carbide inserts. The machining process was carried out as milling and inverse milling with variable parameters: the cutting speed v_c_ = 500–950 m/min, the feed per tooth f_z_ = 0.05–0.29 mm/tooth, the axial depth of cut a_p_ = 3–7.5 mm and the radial depth of cut a_e_ = 6–15 mm. Irrespective of the change in the cutting data, lower values of the roughness parameter Ra, 0.089–0.781 μm, were obtained in inverse milling, otherwise the Ra was in the range 0.93–1.395 μm. The change in the feed per tooth was the factor displaying the most significant impact on the surface finish.

Studies have shown that tool coating should also be considered in the selection of the best machining conditions. This factor was assessed by Muralidharan et al. [12], who attempted to determine the effect of tool coating (carbide tools without coating, with TiN and TiAlN coatings were investigated) on the surface roughness of the Mg-SiC/B_4_C composite material. In the tested cutting conditions, the cutting speed range v_c_ = 18–30 m/min and the feed rate v_f_ = 1200–2000 mm/min, the lowest roughness was obtained when cutting with the TiN tool: Ra = 0.36–0.53 μm. In addition, the increased wear of all tested tools was strongly correlated with the increase in the operational parameters.

In a different study [13], the TiAlN-coated carbide milling cutter was selected to execute the machining of the AZ91D alloy, whose surface was subsequently assessed using the 2D surface and 3D area roughness parameters. The experimental machining involved the change in the cutting parameters in the following ranges: the cutting speed v_c_ = 400–1200 m/min, the feed per tooth f_z_ = 0.05–0.30 mm/tooth, and the axial depth of cut a_p_ = 0.5–6.0 mm. Considering the tested parameters, it was shown that the feed per tooth has the highest impact on the quality of surface finish, whereas the increase in the cutting speed did not significantly affect the 2D parameters, but it had a more visible effect on the 3D area roughness parameters, whose values decreased. The implications emerging from the study suggest that, given the negligible negative effect of the axial depth of cut, machining efficiency may be improved without compromising the quality of the surface finish.

The condition of the surface following milling was also described in the work by Sivam et al. [14]. The study focused specifically on the effects of variable cutting speed, feed per tooth, axial depth of cut, and cutting diameter on the state of the ZE41 alloy surface machined with a multi-edge milling cutter with carbide inserts. The results from the measurements demonstrated that the factors that had the greatest effect on the Ra parameter (1.44–4.11 μm) were the feed per tooth f_z_ = 0.6125–0.645 mm/tooth and the cutting speed v_c_ = 420–720 m/min. Furthermore, the roughness of the component’s surface was shown to increase at higher f_z_ and v_c_. On the other hand, an improvement in the surface quality was observed when machining at the smallest axial depth of cut a_p_ = 1.5 mm and the smallest-diameter tool (Ø20 mm).

Another significant study [15] examined the surface roughness changes in a range of machining parameters: the cutting speed v_c_ = 94–378 m/min, the feed per tooth f_z_ = 0.005–0.08 mm/tooth, and the axial depth of cut a_p_ = 0.5–1.5 mm. Throughout the tested cutting speed values, the Ra parameter of the AZ61 magnesium alloy machined with the milling cutter with carbide inserts was in the range of 0.115–0.403 µm. Low roughness was maintained regardless of the change in the depth of cut and the feed per tooth. The results from the tests indicate that high surface quality is attainable at high efficiency of milling.

The behavior of the AZ61 alloy surface machined with a multi-edge cutter with carbide inserts was also investigated by Chirita et al. [16]. In addition to the operational parameters of milling, the study included two more variables: the feed direction and the cooling method. It was shown that the greatest influence on the surface roughness has the feed per tooth, which was modified in the range of f_z_ = 0.08–0.30 mm/tooth, and the direction of feed. The observed effect of changes in the cutting speed, in the range of v_c_ = 500–1000 m/min, and the axial depth of cut, a_p_ = 0.4–1.6 mm, were shown to only slightly affect the surface roughness of the workpiece. In addition, lower values of the parameter Sa = 0.142–0.795 μm were obtained for the direct feed than for the reverse feed—Sa = 0.205–0.686 μm. Finally, in comparison with dry milling, the application of MQL (minimum quantity lubrication) cooling limited the scatter of results.

The optimal combination of dry milling parameters during the machining of AM60 Mg alloy using a TiN-coated carbide tool was sought by Sathyamoorthy et al. [17]. The set of modified parameters included the cutting speed v_c_ = 30–60 m/min, the feed per tooth f_z_ = 0.025–0.05 mm/tooth and the cutting depth, a_p_ = 0.5–1.5 mm. The tests showed that the condition of the surface deteriorated when the feed per tooth and the depth of cut were increased. That negative effect was offset by increasing the cutting speed: machining at the top rotational speed, feed per revolution f_n_ = 0.1 mm/r and the cutting depth a_p_ = 1 mm, led to obtaining the smallest roughness Ra, of approximately 0.3 µm.

Another Mg alloy, AZ91D, was put to HSM (high speed machining) tests by Ruslan et al. [18]. The face milling cutter, was engaged at a small feed per tooth, 0.03–0.09 mm/tooth; and the axial depth of cut 0.2–0.3 mm, produced a surface characterized by an exceptionally low roughness—Ra = 0.061–0.133 μm. The most advantageous surface finish was obtained at the lowest cutting speed, 900 m/min. As a result, it was confirmed that by providing the high-quality surface finish, milling at high speeds eliminates the need for additional finishing operations, such as grinding or polishing. 

The condition of the machined surface can also be affected by the tool rake angle, as shown by Gziut et al. [19]. Two carbide end mills of a different cutting-edge geometry (γ = 5° and γ = 30°) were used for AZ91HP magnesium alloy milling. The technological parameters that changed during the tests were the cutting speed v_c_ = 400–1200 m/min, the feed per tooth f_z_ = 0.05–0.30 mm/tooth and the axial depth of cut a_p_ = 0.5–3 mm. The 2D roughness parameters, measured on the end face and the lateral face of the samples, showed that a better surface quality was obtained using the tool with a lower tool rake angle (γ = 5°). Additionally, an increase in the cutting speed v_c_ resulted in the decrease in the values of the measured parameters, while an increase in the feed per tooth f_z_ caused their increase. A change in the axial depth of cut a_p_ did not have a significant effect on the surface roughness parameters.

Previous studies have shown the importance of extending the scope of roughness parameters as, in aggregate, they provide a more profound insight into the state and utilitarian features of surfaces, such as their fatigue strength properties. Therefore, this work investigated the 2D roughness parameters (Ra, Rv, Rp, Rt, Rku, Rsk, RSm) and the 3D area roughness parameters (Sa, Sv, Sp, St, Sku, Ssk) to describe the effects of dry milling on the end face and the lateral face of AZ91D magnesium alloys. In addition, the Abbott–Firestone curves of the substrates were determined. The effects of v_c_, f_z_, and a_p_ on selected surface geometry indicators was derived from the experimental and statistical tests. In the future, the method employed in this study could lay the foundation for a decision-making system for a potential application in industry. The novelty of this study consists in that the roughness parameters detailed here will allow the engineers and technologists to infer on the utilitarian and strength features of a surface under consideration. In addition, magnesium alloys are highly innovative materials used by cutting-edge industries, such the aerospace construction.

## 2. Materials and Methods 

### 2.1. Testing Methodology

The primary objective of our study was to determine the impact of the milling parameters on the surface roughness of the machined components. The condition of the surface was described using the end-face and the lateral-face 2D roughness parameters, and 3D area roughness parameters taken on the end face surfaces of the AZ91D magnesium alloy specimens. The tested substrate is one of the most widely used Mg casting alloys and is characterised by very good mechanical properties and resistance to atmospheric corrosion. Casting alloys are primarily found in the aviation and automotive industry applications. The chemical composition of the AZ91D magnesium alloy is given in Table 1.

**Figure 1:**
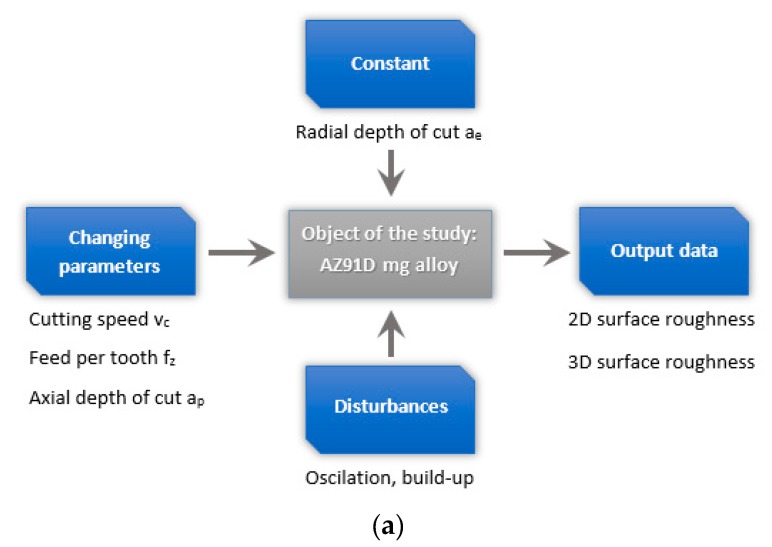

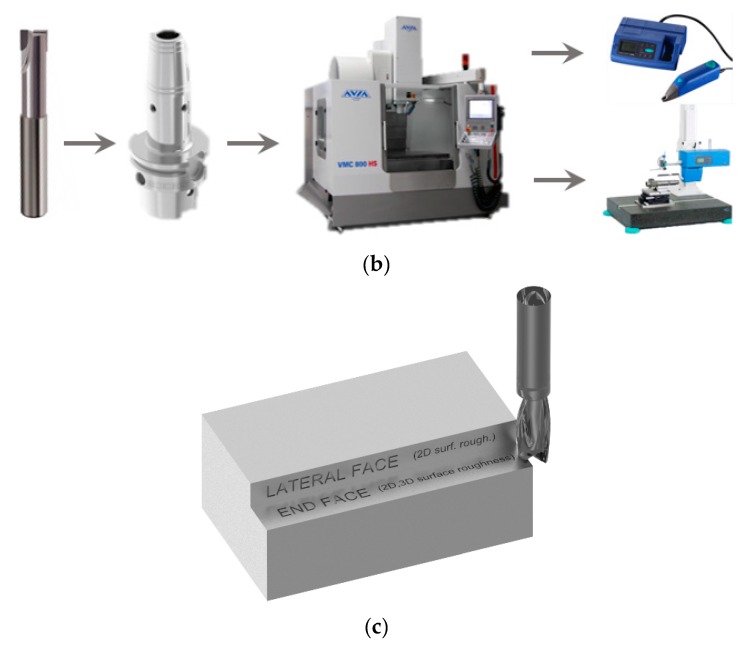
Schematic diagram of (**a**) the test set-up, (**b**) the object of the study and (**c**) milling visualization with the roughness measurement model.

Figure 1 shows the schematics of the study design.

The milling tests were processed by AVIA VMC 800HS (Warsaw, Poland) type vertical machining center, equipped with the Heidenhain iTNC 530 control system, operating at a maximum rotational speed of to 24,000 rev/min. The Ø16 mm double-edge milling cutter with polycrystalline diamond (PCD) inserts, obtained from Guhring (Albstadt, Germany), was chosen for testing, λ_s_ = 0˚. To improve the stability of machining, the milling cutter was mounted in a Shrinkfit SFD tool holder with the HSK-A63 adapter from Seco (Fagersta, Sweden). The tool-holder assembly was balanced dynamically according to ISO 21940-11: 2016 balancing class G 2.5 at 25000 rev/min (the unbalance at 1.14 g mm) by a CIMAT RT 610 balancing machine (Bydgoszcz, Poland). The operational parameters modified during milling were: cutting speed v_c_ = 400–1200 m/min, feed per tooth f_z_ = 0.05–0.30 mm/tooth and axial depth of cut a_p_ = 0.5–6 mm, while the constant parameter was the radial depth of cut a_e_ = 14 mm.

The end-face and the lateral-face 2D roughness parameters were measured by means of a Hommel Roughness Tester T1000 from Hommel Etamic (Jena, Germany) with a Gauss (M1) digital filter, as designated in the International Standard ISO 11562. The specific measurement parameters were: traverse length lt = 4.8 mm and sampling length ln = 0.8 mm, scan rate v_t_ = 0.5 mm/s, and measuring ranges/resolution M = ±320 μm/0.04 μm. Each measurement was performed in 5 repetitions per surface to provide a sufficient amount of data for the calculation of the mean values and the standard deviation of the results. The 3D topography of specimens was assessed on their face surface using the T8000 RC120-400 device from Hommel Etamic (Jena, Germany). The surface scanning area was 1.6 × 1.6 mm at 100 parallel toolpaths, perpendicular to the direction of tool marks.

### 2.2. Statistical Analysis Methodology

The 2D roughness data obtained from the measurements were subjected to statistical verification, intended to test whether the difference between them is statistically significant at the standard significance level α = 0.05. The roughness data were treated as two groups of independent quantitative variables.

In the first stage of the analysis, the Shapiro–Wilk test was employed, testing the normality of distribution

(1) $$\;W=\frac{{\left[\sum_{i}a_{i}\left(n\right)\left(X_{n-i+1}-X_{i}\right)\right]}^{2}}{\sum_{j=1}^{n}{\left(X_{j}-\bar{X}\right)}^{2}}$$

where: $a_{i}$—constant coefficient from tables, $\bar{X}$—mean of the sample.

The obtained quantity was compared with the critical quantity from the tables, W_cr_, to verify whether *W* > W_cr_. Once their normal distribution was confirmed, the hypothesis of equality of variances was tested.

The null hypothesis was

(2) $$H_{0}:\;\sigma_{1}^{2}=\sigma_{2}^{2}$$

The alternative hypothesis was

(3) $$H_{1}:\;\sigma_{1}^{2}\neq \sigma_{2}^{2}$$

The hypothesis was verified using the Fisher–Snedecor *F* distribution test

(4) $$F=\;\frac{\sigma_{I}^{2}}{\sigma_{II}^{2}}$$

where: $\sigma_{I}^{2}$—larger variance, $\sigma_{II}^{2}$—smaller variance.

The degrees of freedom f_1_ and f_2_ were drawn from

(5) $$f_{1}=n_{1}-1$$

(6) $$f_{2}=n_{2}-1$$

where: $\;n_{1}$—sample size 1, $n_{2}$—sample size 2.

The obtained quantity *F* was compared with the critical quantity F_cr_ from the tables. In the case when the hypothesis *F* < F_cr_ was confirmed, the hypothesis of the equality of variances H_0_ was considered confirmed. The null hypothesis of equality of means was verified with the Student’s *t*-test.

The null hypothesis is

(7) $$H_{0}:\;\mu_{1}^{2}=\mu_{2}^{2}$$

and the alternate hypothesis is

(8) $$H_{1}:\;\mu_{1}^{2}\neq \mu_{2}^{2}$$

The *t*-score was calculated using the following formula

(9) $$t=\frac{\bar{X_{1}}-\;\bar{X_{2}}}{\sqrt{\frac{n_{1}\sigma_{1}^{2}+n_{2}\sigma_{2}^{2}}{n_{1}+n_{2}-2}\cdot \left(\frac{1}{n_{1}}+\frac{1}{n_{2}}\right)}}$$

The critical region was determined from the critical value derived from the tables

(10) $$t_{cr}=\left(-\infty ,\;-t_{cr}\rangle \cup \langle t_{cr},\;\infty \right)$$

If the calculated t-score was not in the critical region, then the null hypothesis H_0_ was confirmed, proving that the difference between the means was not statistically significant.

In the case when the null hypothesis was rejected, the Cochran Q test was employed

(11) $$C=\frac{\bar{X_{1}}-\bar{X_{2}}}{\sqrt{\frac{\sigma_{1}^{2}}{n_{1}}+\frac{\sigma_{2}^{2}}{n_{2}}}}$$

In the Cochran Q test, the critical value and the critical region were derived from

(12) $$C_{cr}=\frac{\frac{\sigma_{1}^{2}}{n_{1}-1}t_{1}+\frac{\sigma_{2}^{2}}{n_{2}-1}t_{2}}{\frac{\sigma_{1}^{2}}{n_{1}-1}+\frac{\sigma_{2}^{2}}{n_{2}-1}}$$

(13) $$O_{cr}=\left(-\infty ,\;-C_{cr}\rangle \cup \langle C_{cr},\;\infty \right).\;$$

If the calculated C test statistic was not in the critical region, then the H_0_ hypothesis of the equality of means was confirmed, thus the difference between the means was not statistically significant. All statistical works were carried out using Statistica 12 software [20].

## 3. Results and Discussion

### 3.1. Surface Roughness 2D

**Figure 2:**
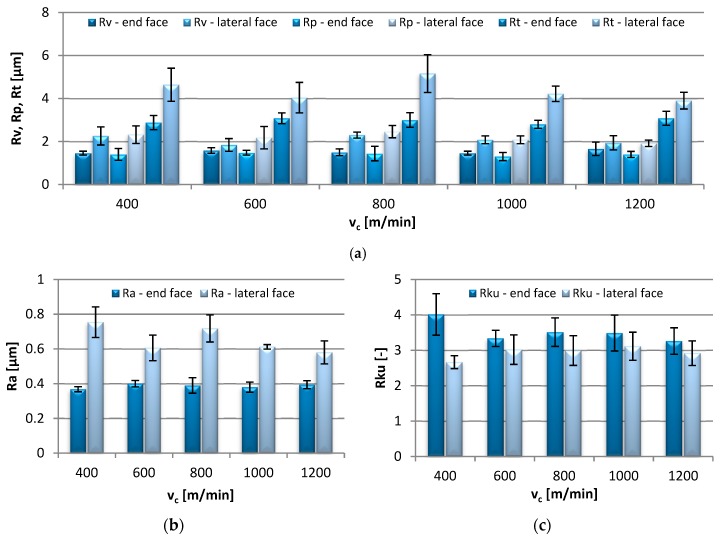

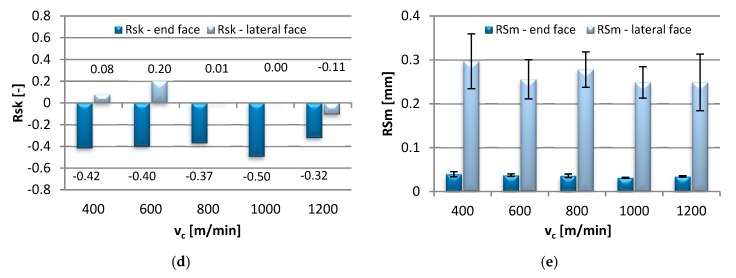
Effect of cutting speed v_c_ change on: (**a**) Rv, Rp, Rt, (**b**) Ra, (**c**) Rku, (**d**) Rsk and (**e**) RSm parameter (f_z_ = 0.15 mm/tooth, a_p_ = 6 mm).

Figure 2 contains bar graphs presenting the influence of the cutting speed change, v_c_ = 400–1200 m/min, on the values of the end-face and lateral-face 2D roughness parameters.

The values of Rv and Rp were comparable and ranged from 1.30–1.66 µm on the end face and 1.84–2.46 µm on the lateral face of the workpiece. Similarly, regarding the Rt parameter, lower values, ranging from 2.80 to 3.08 µm, were also recorded on the end-face surfaces, while on the lateral face, its value ranged from 3.90 to 5.16 µm. The Ra parameter did not exceed 0.40 µm on the end face, similarly as in the study by Kuczmaszewski et al. [7] where the average Ra was reported in the close range (0.3–0.4 µm), while on the lateral surface, its values were significantly higher, varying from 0.58 to 0.75 µm. The results also confirm other previous findings [8,9], where the most beneficial Ra was near 0.4 μm and was obtained by modifying the cutting speed. Among the surface roughness parameters, only the end-face Rku was higher in comparison with the value on the lateral face of the specimens. Kurtosis values greater than 3, as here, designate a surface with sharp peaks and valleys, which in the case of mating surfaces contributes to reducing the friction coefficient; on the lateral face, the peaks had normal distribution (values close to 3). The negative values of the end-face Rsk are indicative of flat-topped distribution, while the values close to zero on the lateral face indicate a symmetrical distribution of peaks. For clarity, the numerical values of the Rsk parameter are presented in the graph. The RSm values on the end face were in the range of 0.031–0.039 mm, and were several times lower than those recorded on the lateral face of the specimens, 0.249–0.297 mm. The change in the cutting speed was not found to significantly impact any of the 2D parameters.

**Figure 3:**
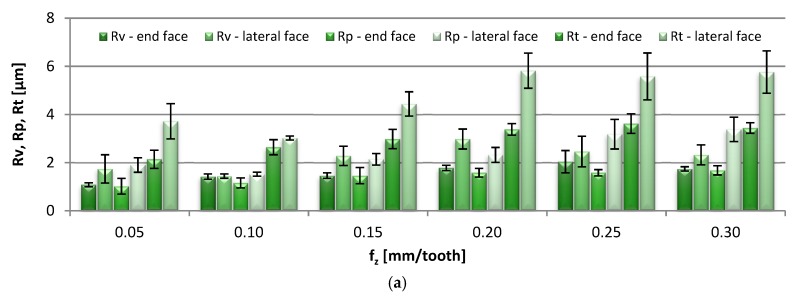

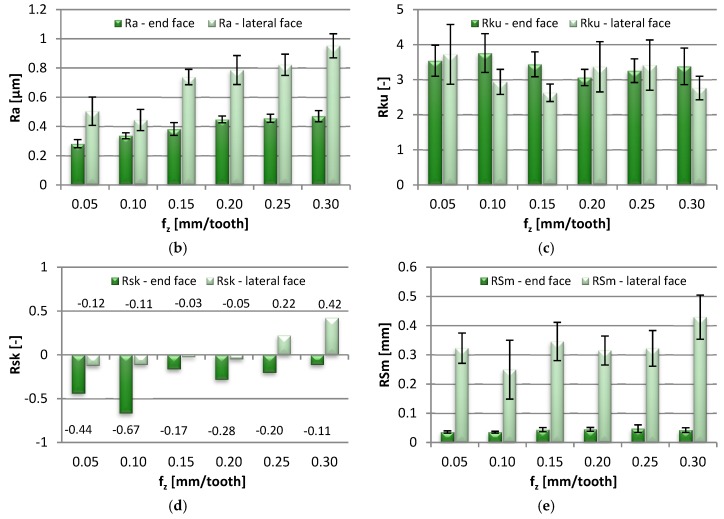
Effect of feed per tooth f_z_ change on: (**a**) Rv, Rp, Rt, (**b**) Ra, (**c**) Rku, (**d**) Rsk and (**e**) RSm parameter (v_c_ = 800 m/min, a_p_ = 6 mm).

The bar charts in Figure 3 represent the effects that the feed per tooth adjustment, f_z_ = 0.05–0.30 mm/tooth, had on the end-face and the lateral-face 2D surface roughness parameters. 

Comparable values of Rv and Rp were obtained on the end face of the specimens, amounting to 1.02–2.04 µm. The lateral-face parameters were notably higher, in the range of 1.44–3.38 µm. Although at higher feed per tooth rates both parameters tended to increase, it was Rv that showed a stronger response to such adjustments. A similar effect was observed in the case of the Rt parameter, which raised to 2.14–3.62 µm on the end face, and 3.02–5.82 µm on the lateral face of the specimens. Similarly, an increase in the feed per tooth triggered an increase in the Ra parameter, which on the end face amounted to 0.28–0.0.47 µm (whereas in a former study [7] the parameters were 0.2–0.5 µm) and on the lateral face to 0.44–0.95 µm. Similar Ra results were also reported in analogous studies [8,9], where the Ra in the range of 0.2 µm to approximately 0.75 µm was produced by cutting with a feed per revolution of 0.05–0.4 mm/rev. The end-face Rku parameter was reported to exceed the value of 3 in the entire range of tested feeds, which is characteristic of surfaces with sharp peaks and valleys; the kurtosis values on the lateral face oscillated in the region of 3, thus indicating the normal peak distribution. The Rsk parameter on the end face was negative in the tested range of feed per tooth changes, which is indicative of a flat-topped distribution of the profile heights, while the lateral-face Rsk was variable, fluctuating between negative and positive. For easier interpretation, the numerical values of the Rsk are included in the graph. In the case of the RSm parameter, on the end face it ranged from 0.035 to 0.047 mm, which was a several-fold lower value compared with the measurement results on the lateral face of the specimens: 0.249–0.429 mm.

**Figure 4:**
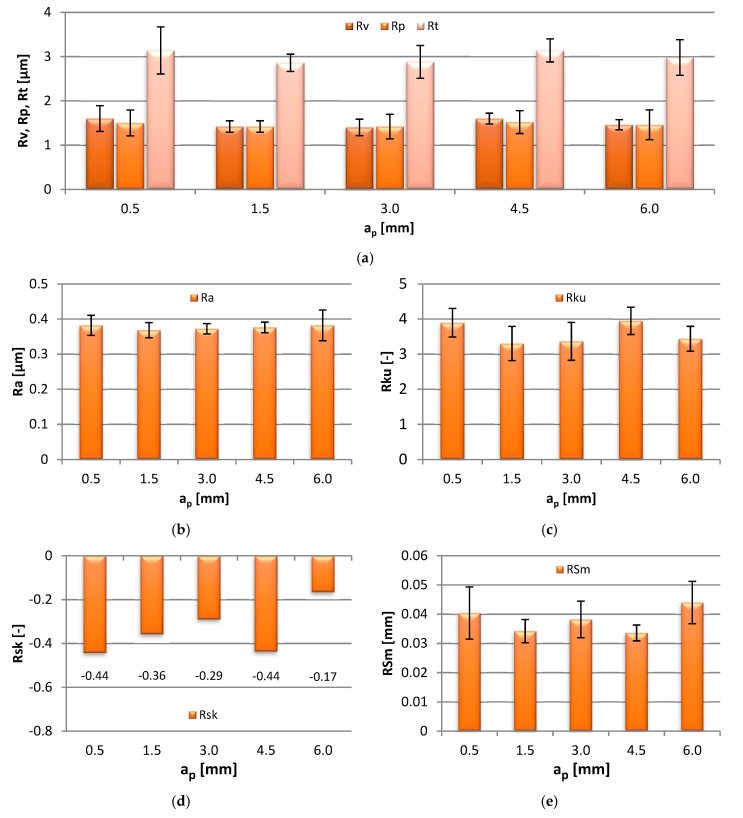
Effect of axial depth of cut a_p_ change on: (**a**) Rv, Rp, Rt, (**b**) Ra, (**c**) Rku, (**d**) Rsk and (**e**) RSm parameter (v_c_ = 800 m/min, f_z_ = 0.15 mm/tooth).

The impact of the axial depth of cut changes, in the range of a_p_ = 0.5–6 mm, on the 2D roughness parameters of the specimens is shown in the bar graphs in Figure 4.

The values of Rv and Rp amounted to 1.42–1.60 µm, which was constant throughout the range of a_p_ applied during milling. A similar consistency of results was observed in the case of the Rt parameter, 2.86–3.14 µm. Similarly, a negligible effect of the rising axial depth of cut on the Ra parameter was observed. The parameter readings were repeatedly constant at 0.37–0.38 µm, which confirms previous findings [8,9]—for a_p_ = 0.1–0.5 mm the average Ra was near 0.4 µm. Regardless of the modification of the axial depth of cut during machining, the workpiece surface on the end face was characterized by sharp peaks and valleys, which emerges from Rku > 3. The Rsk > 0 was observed in the entire range of a_p_, indicating that the surface heights were evenly distributed. The RSm parameter was in the range of 0.034–0.044 mm, and did not show a dramatic change in response to the increase in the axial depth of cut. None of the 2D surface roughness parameters was strongly affected by the change in the axial depth of cut during milling.

### 3.2. Surface Roughness 3D

**Figure 5:**
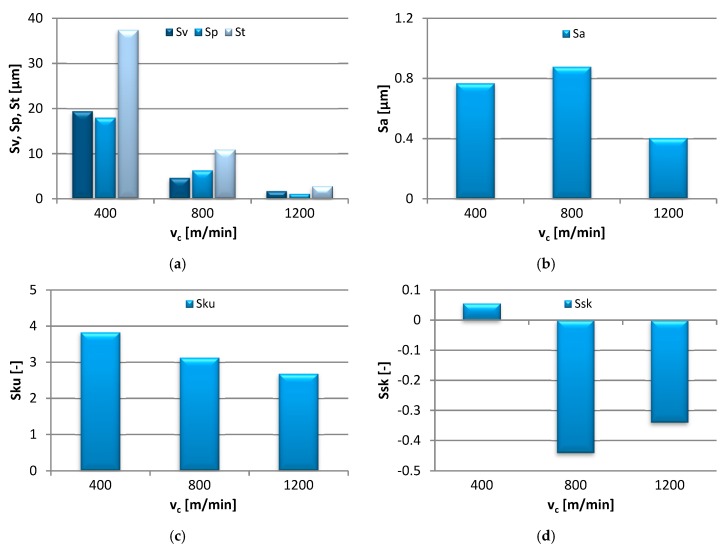
Effect of cutting speed v_c_ change on: (**a**) Sv, Sp, St, (**b**) Sa, (**c**) Sku and (**d**) Ssk parameter (f_z_ = 0.15 mm/tooth, a_p_ = 6 mm).

The effects of the cutting speed adjustment in the range of v_c_ = 400–1200 m/min on the 3D area roughness parameters measured on the end face of the specimen are shown in Figure 5.

The values of Sv, Sp, and St showed a gradual decrease with the elevating cutting speed, i.e., from 19.40 µm to 1.69 µm, from 18.00 µm to 1.10 µm, and from 37.40 µm to 2.79 µm respectively. Following a slight increase at the medium cutting speed, v_c_ = 800 m/min, the Sa eventually dropped from the initial 0.77 µm to 0.40 µm. A similar effect was observed in the Sku, which decreased from 3.83 to 2.68, thus indicating that initially sharp, the peaks and valleys became more rounded as the cutting speed progressed. With the increasing cutting speed, the value of Ssk dropped below 0, denoting flat surfaces with scattered peaks.

**Figure 6:**
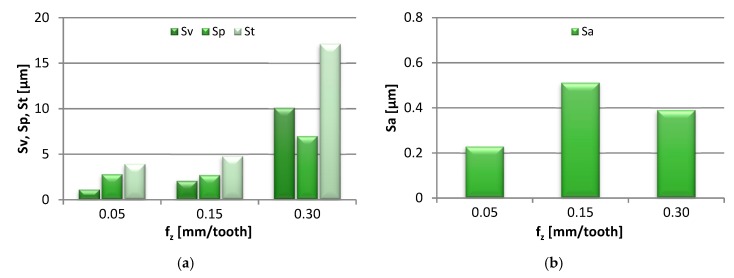

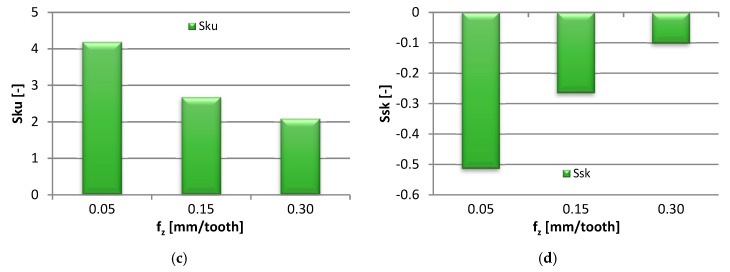
Effect of feed per tooth f_z_ change on: (**a**) Sv, Sp, St, (**b**) Sa, (**c**) Sku and (**d**) Ssk parameter (v_c_ = 800 m/min, a_p_ = 6 mm).

Figure 6 presents the effect of increasing the feed per tooth, from 0.05 to 0.30 mm/tooth, on the end-face 3D area roughness parameters of the specimens. The results are shown as bar graphs. 

Sv and St were shown to increase gradually, respectively from 1.13 to 10.10 µm and from 3.92 to 17.10 µm. The values of Sp were eventually observed to increase from 2.70 to 6.99 µm, after a slight drop in value at f_z_ = 0.15 mm/tooth. As a result of the change in the feed per tooth, they initially increased from 0.23 to 0.51 µm, and dropped at the highest f_z_ setting. Regarding the Sku parameter, its value showed a linear decrease from 4.19 to 2.09, indicating that the surface heights became progressively rounded with the rising f_z_. In turn, the Ssk parameter was found to assume negative values, which is explained by the change from a flat-topped to a more symmetrical height distribution. 

**Figure 7:**
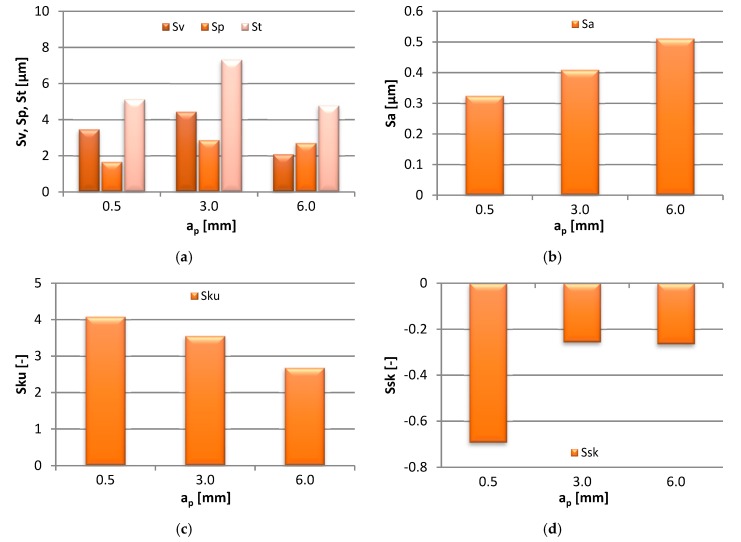
Effect of axial depth of cut a_p_ change on: (**a**) Sv, Sp, St, (**b**) Sa, (**c**) Sku and (**d**) Ssk parameter (v_c_ = 800 m/min, f_z_ = 0.15 mm/tooth).

The bar charts in Figure 7 represent the effect that the modification in the axial depth of cut (a_p_ = 0.5–6 mm) had on the end-face 3D area roughness characteristics of the specimen surface. 

As shown above, the Sv, Sp, and St all responded positively to the increase in a_p_ to 3.0 mm and decreased with the further increase in the cutting depth. The change in Sv was in the range 2.08–4.44 µm, Sp—1.66–2.87 µm, and St—4.78–7.31 µm. With progressing a_p_, Sa was reported to increase linearly from 0.32 to 0.51 µm. The kurtosis parameter, Sku, gradually decreased from 4.08 to 2.68, i.e. the changes in the machining parameters resulted in the rounding of the sharp surface peaks. A decrease in the value of Ssk below 0, increasing slightly at higher a_p_, indicated that the surface acquired the flat-topped distribution of heights.

Table 2 contains surface topography maps of the end face of the specimen and Abbott–Firestone curves determined during milling at extreme cutting speeds, v_c_ = 400 m/min and v_c_ = 1200 m/min.

From the data presented in Table 2, it can be seen that with the progressing cutting speed, the number of heights increased while the peaks became rounded, which can be inferred from the drop in kurtosis below 3. This observation is further confirmed by the shape of the Abbot–Firestone curve, which shifted from the s-shaped degressive to degressive-progressive, and thus could be indicative of the improvement in the wear resistance of the surface. The increase in the v_c_ parameter of milling was, furthermore, found to increase the valley depth, which is typical of flat-topped surfaces, when the skewness is negative. 

The end-face surface topography maps and the Abbott-Firestone curves in Table 3 show the condition of the surface following milling with the lowest and the highest feed per tooth settings, f_z_ = 0.05 mm/tooth and f_z_ = 0.30 mm/tooth.

With the progressing feed per tooth, the sharp peaks become increasingly rounded, as the kurtosis is reduced below 3. In addition, the initially flat-topped distribution became more symmetrical. Irrespective of the change in the feed per tooth, the Abbott–Firestone curve remained degressive–progressive.

Presented in Table 4 are the surface topography maps and the bearing area curves corresponding to the milling with the lowest (a_p_ = 0.5 mm) and the highest axial depth of cut (a_p_ = 6 mm).

As a result of the increase in a_p,_ the sharp height peaks became rounded, as confirmed by the kurtosis value lower than 3. As the skewness values approached 0, the height distribution changed from the flat topped to a more symmetrical one. The Abbott–Firestone curve levelled off slightly, however, it retained a degressive–progressive shape.

### 3.3. Statistical Analysis

**Figure 8:**
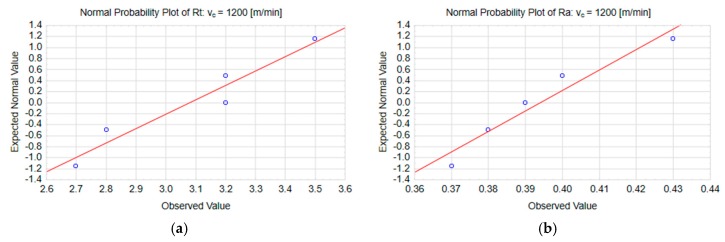
Normal probability plots for the parameters: (**a**) Rt and (**b**) Ra at the cutting speed v_c_ = 1200 m/min.

The normal probability plots in Figure 8 show a typical distribution of Rt and Ra on the end face of the workpiece surface machined with the highest cutting speed, v_c_ = 1200 m/min.

The results from the Shapiro–Wilk test of normality are shown in Table 5. The test concerned the distribution of the end-face 2D surface roughness parameters of the specimens subjected to milling at the variable cutting speed v_c_. The reported results were shown to have normal distribution in all considered cases. 

Table 6 presents the results from the statistical analysis of the end-face 2D surface roughness parameters of the specimens subjected to milling with the variable cutting speed v_c_. The lowest (v_c_ = 400 m/min) and the highest speeds (v_c_ = 1200 m/min) were considered. 

From the analysis, it can be seen that the equality of variances hypothesis was confirmed in all parameters except for Rsk and RSm. The hypothesis assuming the equality of means at α = 0.05 was confirmed with no exceptions.

**Figure 9:**
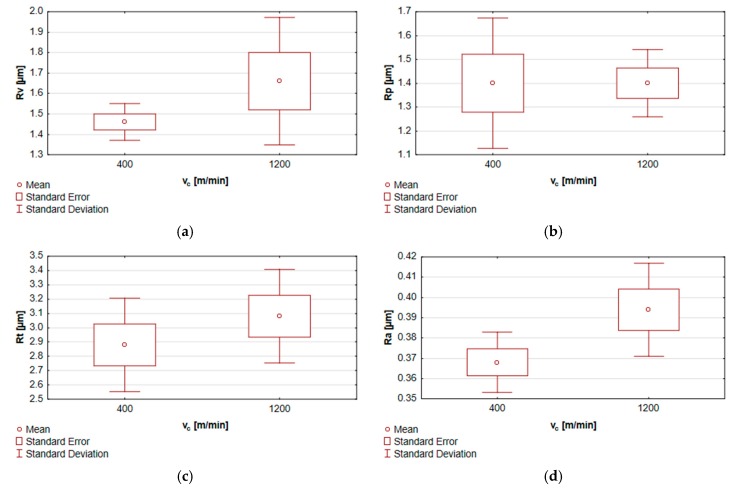

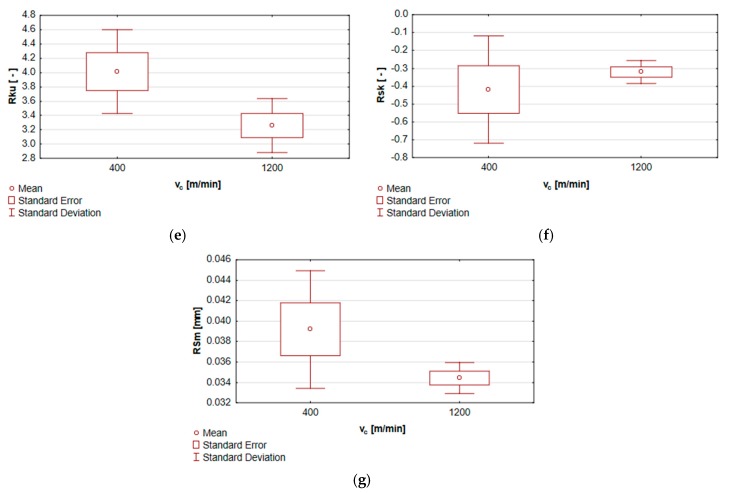
Tests of equality of means for the end face of specimens machined at the variable cutting speed v_c_ for the parameters: (**a**) Rt, (**b**) Rv, (**c**) Rp, (**d**) Ra, (**e**) Rsk, (**f**) Rku and (**g**) RSm.

Figure 9 contains the box and whisker graphs accounting for the results from the tests of equality of means describing the end-face 2D surface roughness parameters of the specimens subjected to milling with varying cutting speeds.

**Figure 10:**
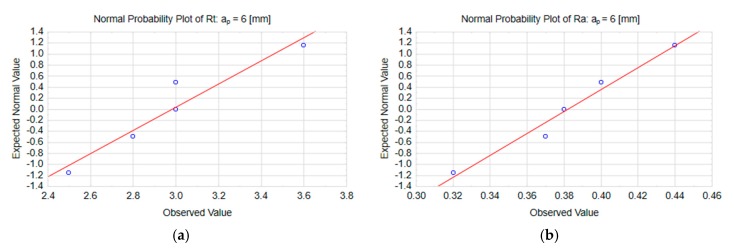
Normal probability plots for the parameters: (**a**) Rt and (**b**) Ra at the axial depth of cut a_p_ = 6 mm.

Figure 10 shows the normal probability plots for the results of Rt and Ra measurements carried out on the end face of the specimens following milling with the maximal axial depth of cut a_p_ = 6 mm.

The results from the Shapiro–Wilk test of normality are shown in Table 7. The tests concerned the values of the end-face 2D surface roughness parameters of the workpiece following milling with the variable axial depth of cut a_p_. In all tested variants, the results had normal distribution. 

The results from the statistical analysis of the end-face 2D surface roughness parameters of the specimens machined with various axial depths of cut a_p_ are given in Table 8; the lowest (a_p_ = 0.5 mm) and the highest (a_p_ = 6 mm) axial depths of cut are compared.

Statistical analysis confirmed the equality of variance hypothesis for all the parameters except for Rv. The equality of means hypothesis at α = 0.05 was confirmed with no exceptions.

**Figure 11:**
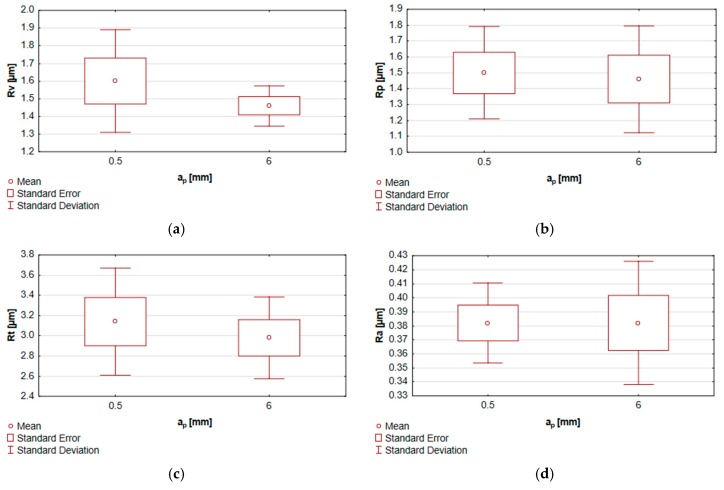

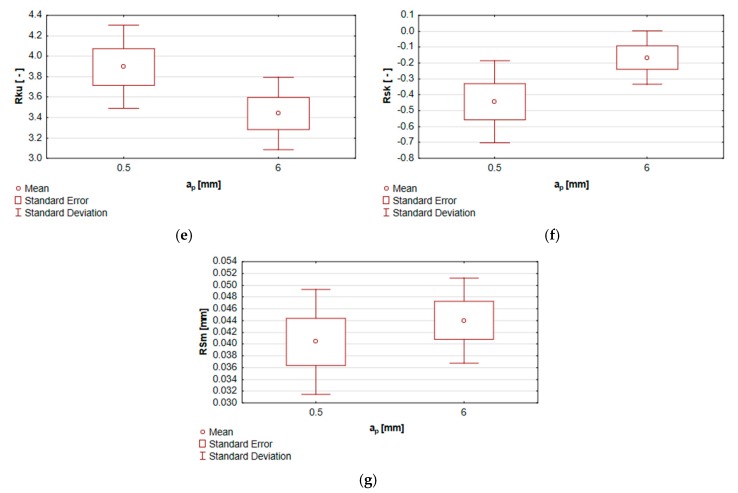
Tests of equality of means for the end face of specimens machined at the variable axial depth of cut a_p_ for the parameters: (**a**) Rt, (**b**) Rv, (**c**) Rp, (**d**) Ra, (**e**) Rsk, (**f**) Rku and (**g**) RSm.

The results from the tests of equality of means describing the end-face 2D surface roughness of specimens subjected to milling with the variable axial depth of cut a_p_ are presented in Figure 11, in the form of the box and whisker graphs.

The analyzed data clearly indicate that the changes in the cutting speed v_c_ and the axial depth of cut a_p_ have had no statistically significant impact on the condition of the workpiece surface, expressed by the end-face 2D roughness parameters.

## 4. Conclusions

Several conclusions emerge from the experimental and statistical test results reported in this study:

- The modification of the cutting speed v_c_ and the axial depth of cut a_p_ settings did not significantly affect the end-face/lateral-face 2D surface roughness parameters;
- The modification of the feed per tooth f_z_ has the greatest impact on the condition of the surface among the modified operational parameters: its increase correlates with the linear increase in the value of most of the 2D surface roughness parameters;
- Lower values of the end-face 3D area roughness parameters were observed in milling with the highest cutting speed (v_c_ = 1200 m/min);
- The cutting speed v_c_ and the axial depth of cut a_p_ had a negligible effect on the mean values of the end-face 2D surface roughness parameters;
- The statistical analysis of the end-face 2D roughness parameters after milling with the variable cutting speed v_c_ confirmed the equality of variance in most of the cases (except for Rsk and RSm), and, in milling with the variable axial depth of cut a_p_, it was confirmed in all cases except the Rv parameter;
- The weak correlation between the change in the axial depth of cut a_p_ and the roughness parameters of the surface implies that, by increasing the a_p_, the efficiency of milling may be improved without exerting a negative effect on the quality of the surface of components.

## Figures and Tables

**Table 1:** Chemical composition (wt %) of AZ91D magnesium alloy.

Chemical Composition	Al	Zn	Mn	Si	Cu	Fe	Ni	Be	Mg
AZ91D	8.91	0.66	0.22	0.016	0.002	0.002	0.001	0.001	rest/other

**Table 2:** Surface topography maps and Abbott–Firestone curves obtained with variable cutting speeds.

Surface Topography	Abbott–Firestone Curve
v_c_ = 400 m/min; f_z_ = 0.15 mm/tooth; a_p_ = 6 mm	
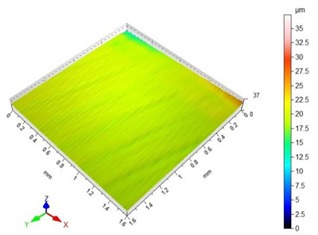	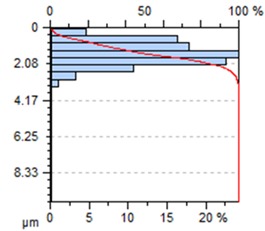
v_c_ = 1200 m/min; f_z_ = 0.15 mm/tooth; a_p_ = 6 mm	
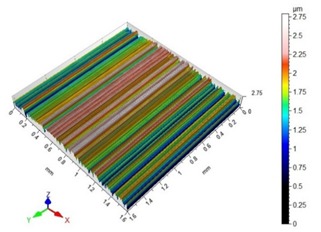	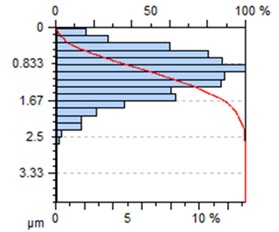

**Table 3:** Surface topography maps and Abbott–Firestone curves at the variable feed per tooth.

Surface Topography	Abbott–Firestone Curve
v_c_ = 800 m/min; f_z_ = 0.05 mm/tooth; a_p_ = 6 mm	
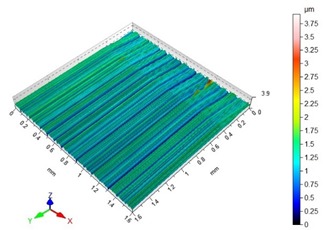	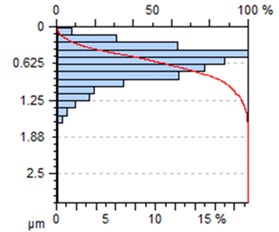
v_c_ = 800 m/min; f_z_ = 0.30 mm/tooth; a_p_ = 6 mm	
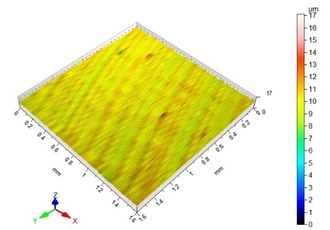	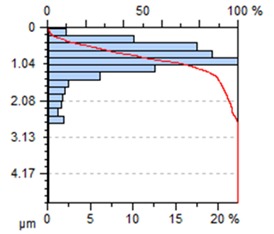

**Table 4:** Surface topography maps and Abbott–Firestone curves at the variable axial depth of cut.

Surface Topography	Abbott–Firestone Curve
v_c_ = 800 m/min; f_z_ = 0.15 mm/tooth; a_p_ = 0.5 mm	
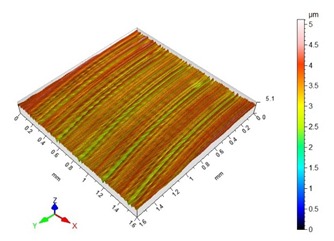	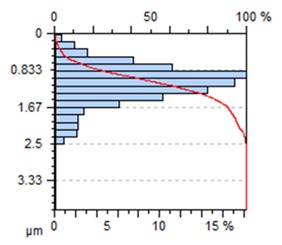
v_c_ = 800 m/min; f_z_ = 0.15 mm/tooth; a_p_ = 6 mm	
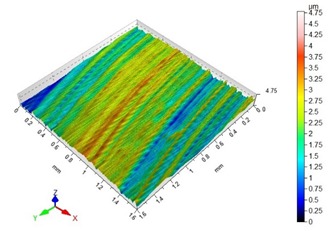	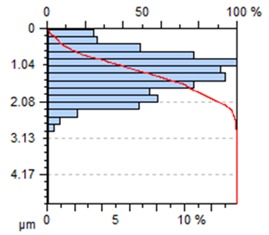

**Table 5:** Results from the normality tests at the variable cutting speed v_c_.

v_c_ (m/min)	Rv	Rp	Rt	Ra	Rku	Rsk	RSm	Critical Value
400	0.771	0.964	0.922	0.956	0.942	0.949	0.823	0.762
1200	0.788	0.767	0.922	0.943	0.833	0.887	0.768	

**Table 6:** Statistical analysis of surface roughness parameters at the variable cutting speed v_c_.

				Equality of Variances Hypothesis			Equality of Means Hypothesis				
Par.	v_c_ (m/min)	$\bar{X}$	σ^2^	F Statistic Value	F Critical Value	Conclusion	C Statistic Value	C Critical Value	t Statistic Value	t Critical Value	Conclusion
Rv	400	1.46	0.0080	0.0816	6.3883	Equal	-	-	1.229	2.776	Equal
	1200	1.66	0.0980								
Rp	400	1.40	0.0750	3.7500	6.3883	Equal	-		0.000	2.776	Equal
	1200	1.40	0.0200								
Rt	400	2.88	0.1070	1.0000	6.3883	Equal	-	-	0.865	2.776	Equal
	1200	3.08	0.1070								
Ra	400	0.37	0.0002	0.4151	6.3883	Equal	-	-	1.899	2.776	Equal
	1200	0.39	0.0005								
Rku	400	4.01	0.3447	2.4508	6.3883	Equal	-	-	−2.162	2.776	Equal
	1200	3.26	0.1406								
Rsk	400	−0.42	0.0904	20.9336	6.3883	Unequal	0.637	2.776	-	-	Equal
	1200	−0.32	0.0043								
RSm	400	0.04	0.0000	14.4348	6.3883	Unequal	1.611	2.776	-	-	Equal
	1200	0.03	0.0000								

**Table 7:** Statistical analysis of surface roughness parameters at the variable axial depth of cut a_p_.

a_p_ (mm)	Rv	Rp	Rt	Ra	Rku	Rsk	RSm	Critical Value
0.5	0.928	0.928	0.857	0.804	0.908	0.958	0.857	0.762
6	0.961	0.925	0.937	0.985	0.927	0.950	0.919	

**Table 8:** Statistical analysis of surface roughness parameters at the variable axial depth of cut a_p_.

				Equality of Variances Hypothesis			Equality of Means Hypothesis				
Par.	a_p_ (mm)	$\bar{X}$	σ^2^	F Statistic Value	F Critical Value	Conclusion	C Statistic Value	C Critical Value	t Statistic Value	t Critical Value	Conclusion
Rv	0.5	1.60	0.0850	6.5385	6.3883	Unequal	0.894	2.776	-	-	Equal
	6	1.46	0.0130								
Rp	0.5	1.50	0.0850	0.7522	6.3883	Equal	-	-	−0.180	2.776	Equal
	6	1.46	0.1130								
Rt	0.5	3.14	0.2830	1.7469	6.3883	Equal	-	-	−0.480	2.776	Equal
	6	2.98	0.1620								
Ra	0.5	0.38	0.0008	0.4271	6.3883	Equal	-	-	0.000	2.776	Equal
	6	0.38	0.0019								
Rku	0.5	3.90	0.1660	1.3211	6.3883	Equal	-	-	−1.692	2.776	Equal
	6	3.44	0.1257								
Rsk	0.5	−0.44	0.0682	2.4352	6.3883	Equal	-	-	1.773	2.776	Equal
	6	−0.17	0.0280								
RSm	0.5	0.04	0.0001	1.5105	6.3883	Equal	-	-	0.627	2.776	Equal
	6	0.04	0.0001								
